# Fungal Levels in the Home and Allergic Rhinitis by 5 Years of Age

**DOI:** 10.1289/ehp.7844

**Published:** 2005-05-20

**Authors:** Paul C. Stark, Juan C. Celedón, Ginger L. Chew, Louise M. Ryan, Harriet A. Burge, Michael L. Muilenberg, Diane R. Gold

**Affiliations:** 1 Biostatistics Research Center, Institute for Clinical Research and Health Policy Studies, Department of Medicine, Tufts–New England Medical Center, Boston, Massachusetts, USA; 2 Channing Laboratory, Department of Medicine, Brigham and Women’s Hospital and the Harvard Medical School, Boston, Massachusetts, USA; 3 Department of Environmental Health Sciences, Mailman School of Public Health, Columbia University, New York, New York, USA; 4 Department of Biostatistics, and; 5 Department of Environmental Health, Harvard University School of Public Health, Boston, Massachusetts, USA

**Keywords:** allergic rhinitis, fungi, mold, respiratory health effects, water damage

## Abstract

Studies have repeatedly demonstrated that sensitization to fungi, such as *Alternaria*, is strongly associated with allergic rhinitis and asthma in children. However, the role of exposure to fungi in the development of childhood allergic rhinitis is poorly understood. In a prospective birth cohort of 405 children of asthmatic/allergic parents from metropolitan Boston, Massachusetts, we examined in-home high fungal concentrations (> 90th percentile) measured once within the first 3 months of life as predictors of doctor-diagnosed allergic rhinitis in the first 5 years of life. In multivariate Cox regression analyses, predictors of allergic rhinitis included high levels of dust-borne *Aspergillus* [hazard ratio (HR) = 3.27; 95% confidence interval (CI), 1.50–7.14], *Aureobasidium* (HR = 3.04; 95% CI, 1.33–6.93), and yeasts (HR = 2.67; 95% CI, 1.26–5.66). The factors controlled for in these analyses included water damage or mild or mildew in the building during the first year of the child’s life, any lower respiratory tract infection in the first year, male sex, African-American race, fall date of birth, and maternal IgE to *Alternaria* > 0.35 U/mL. Dust-borne *Alternaria* and non-sporulating and total fungi were also predictors of allergic rhinitis in models excluding other fungi but adjusting for all of the potential confounders listed above. High measured fungal concentrations and reports of water damage, mold, or mildew in homes may predispose children with a family history of asthma or allergy to the development of allergic rhinitis.

Studies have repeatedly demonstrated that sensitization to fungi, such as *Alternaria*, is strongly associated with allergic rhinitis and asthma in children (Arshad et al. 2001; Downs et al. 2001; Halonen et al. 1997; Nolles et al. 2001). However, the role of exposure to fungi, measured in environmental samples, in the development of childhood allergic rhinitis is poorly understood. Allergic rhinitis affects an estimated 20–40 million people in the United States alone, and the incidence is increasing. Allergic rhinitis has been associated with snoring, sleep apnea, and sleep disturbances in children (Camhi et al. 2000; Corbo et al. 2001; Scharf and Cohen 1998). The prevalence of symptoms of allergic rhinoconjunctivitis ranged between 15 and 25% among children 13–14 years of age who lived in the United States or the United Kingdom (International Study of Asthma and Allergies in Childhood 1998). A recent study documented that 93% of adolescents with allergic asthma also have allergic rhinitis. The diagnosis of allergic rhinitis preceded asthma in up to 64% of patients with both diseases (Kapsali et al. 1997). Because allergic rhinitis is a common disease that lessens quality of life and is associated with significant morbidity, determining environmental exposures associated with the development of allergic rhinitis is of great public health importance.

In a prospective birth-cohort study of children with parental history of asthma or allergies, we assessed environmental fungal exposure (culturable fungi in house air and dust samples) and evaluated whether high fungal levels were independently associated with doctor-diagnosed allergic rhinitis in the first 5 years of life.

## Materials and Methods

### Study protocol.

Participants were part of a metropolitan Boston, Massachusetts, prospective birth-cohort study designed to examine relationships between amounts of indoor allergens, including fungi, and the development of allergy and asthma. The study was approved by the institutional review board of Brigham and Women’s Hospital. With the consent of the primary caregiver, screening and recruitment of the families, which we have previously described (Gold et al. 1999), was conducted between September 1994 and June 1996. Monday through Friday, all mothers who delivered at Brigham and Women’s Hospital, a large Boston hospital, were approached for screening within 24–48 hr after delivery. Eligibility criteria included residence inside route 128 (a highway encircling the Boston metropolitan area); maternal age ≥18 years; history of hay fever, asthma, or allergies in either parent; and maternal ability to speak English or Spanish. Families were not screened if the newborn was hospitalized in the intensive care unit, was of gestational age < 36 weeks, or had a congenital anomaly. One month after birth, a questionnaire regarding the child’s health was administered by telephone to families who had initially expressed interest in participating in the study and who fit the inclusion criteria. At 1 month, parents who expressed plans to move or lack of interest in the study were also excluded. Of the 1,405 families screened, 906 were excluded from the study before the initial home visit. Reasons for exclusion were reluctance to participate in a longitudinal study (51%), plans to move within 1 year (39%), early loss to follow-up (9%), and other (1%). After written informed consent was obtained, a home visit was made when the child was 2–3 months of age, and a questionnaire regarding health outcomes, home characteristics, environmental exposures, smoking, and demographics was administered by trained research assistants. Every 2 months, beginning when the child was 2 months old and continuing until the second birthday, a telephone questionnaire was administered to the child’s primary caretaker. After that, health outcomes were ascertained semiannually.

At the initial home visit, indoor air samples were collected from each home using a Burkard culture plate sampler (Burkard Manufacturing Co. Ltd., Hertfordshire, UK) operated at 45 L/min [calculated cut point, diameter allowing 50% (D_50_) = 2 μm] that collected particles onto DG18 agar (a glucose medium containing dichloran and 18% glycerol) in 90-mm Petri dishes. Sequential duplicate 1-min air samples were collected in the bedroom 1–1.5 m above the area of the floor demarcated for dust collection. After sampling, the Petri plates were returned to the laboratory on the same day for incubation. The sampler sieve plate was cleaned with an isopropanol swab after each home visit.

After air samples were collected, 2 m^2^ of the floor surrounding the newborn’s bed was vacuumed for 5 min, using a Eureka Mighty-Mite canister vacuum cleaner (Eureka Co., Bloomington, IN) modified to collect dust in a 19- × 90-mm cellulose extraction thimble (Whatman International Ltd., Herefordshire, UK). In cases where both a smooth floor and a rug were present, 2.5 min was devoted to sampling the rug and 2.5 min was spent vacuuming the smooth floor surface. After sampling, the thimble was sealed in a plastic bag and returned to the laboratory for sifting (425-μm mesh sieve) and weighing.

A weighed aliquot of fine dust from the dust sampling was suspended (~ 25 mg/mL) and serially diluted (e.g., full strength, 1:10, and 1:100) in an aqueous solution of 0.02% Tween. Duplicates of each dilution were spread-plated on DG18 culture media (Verhoeff et al. 1994). After a mean ± SD incubation period of 27.9 ± 14.5 days, we identified fungal colonies on both air- and dust-sample plates to genus (subgenus in the case of *Aspergillus* isolates) using standard mycologic criteria (Ellis 1971; von Arx 1970). We adjusted the number of colonies recovered on the air sample plates for possible multiple impactions using the positive-hole correction equation (Andersen 1958). The calculated concentrations of dust-borne fungi were the number of colony-forming units (cfu) per gram of dust, and those of airborne fungi were cfu per cubic meter of air. We prioritized the use of limited amounts of dust collected from a home so that measurement of allergens for cockroach, cat, and dust mites could be performed first (Chew et al. 1998), followed by analyses of fungi and endotoxin, respectively. If there was not enough dust for allergen analyses, then the fungal analyses were performed first. Thus, when there was > 200 mg or < 25 mg dust collected, fungal concentrations were determined. There were 84 children eliminated because the amount of dust collected was between 25 and 200 mg.

### Fungal data.

In previous analyses of these data (Stark et al. 2003), we found that it was useful to create indices indicating high levels of fungi in the home. The variable was coded 1 if that home produced “high” levels of that fungus, and 0 otherwise. We define “high” as greater than the 90th percentile for each taxon. Based on Akaike’s Information Criterion (Hastie and Tibshirani 1990) and –2 log likelihood, models with indices for high fungal levels fit the data better than do models treating the fungal concentrations as continuous or natural logarithm–transformed data.

### Definition of allergic rhinitis and predictor variables.

We assessed allergic rhinitis from 12 to 60 months of age by asking “Has your child had allergic rhinitis or hay fever diagnosed by a doctor?” Children without 12-month follow-up (*n* = 10) were excluded, leaving 405 children with > 12 months of health outcome data, as well as fungal data.

### Definitions of other predictor variables.

Sociodemographic, familial, and perinatal variables considered for inclusion in the multivariate analysis of the relation between indoor fungal exposure and allergic rhinitis included sex, season of birth [defined as winter (December–February), spring (March–May), summer (June–August), or fall (September–November)], race/ethnicity of the child according to parental reporting (Litonjua et al. 1998), total annual family income (< $30,000, $30,000–50,000, and > $50,000), active maternal history of asthma (physician-diagnosed asthma and wheeze in the past year), maternal sensitization to *Alternaria* [specific immunoglobulin E (IgE) > 0.35 U/mL], maternal smoking during pregnancy, and presence of older siblings in the household.

Additional variables considered for inclusion in the multivariate analysis included report of any of the following in the child’s first year of life: any physician-diagnosed lower respiratory tract infections (pneumonia, croup, bronchitis, or bronchiolitis), as determined by bimonthly questions to the child’s primary caregiver (Celedon et al. 1999); visible water damage; mold or mildew inside the home; and report of water damage in the basement, presence of concrete floors in the baby’s room, use of a dehumidifier, and use of an air conditioner. A composite variable representing the presence of either water damage or visible mold or mildew in the home in the child’s first year of life was also examined.

### Statistical methods.

All final models reported used survival analysis (Cox proportional hazards regression) to explore time to first report of doctor-diagnosed allergic rhinitis or hay fever. We performed initial exploratory analyses of univariate associations between allergic rhinitis and potential predictor variables using logistic regression, chi-square analyses, and Cox regression. We performed all statistical analyses using SAS statistical software (version 8.2, SAS Institute Inc., Cary, NC) and S-Plus (version 6.1, Insightful Corporation, Seattle, WA). Multivariate models were constructed that included significant (*p* ≤0.05) fungal taxa, significant independent predictors of allergic rhinitis, and variables that may confound the relationship between the fungal levels and allergic rhinitis. The proportional hazards assumption was validated in final models.

### Lasso approach.

Multivariate models were independently validated using the lasso approach for model shrinkage described by Tibshirani (1997). This technique prevents the tendency to overfit a model by constraining the effect that the variables will have in the model. This is one approach used to combat the multiple-comparison hazards that arise when so many different taxa of fungi need to be considered.

## Results

Of the 405 children included in this analysis, 52 (12.8%) were diagnosed with allergic rhinitis or hay fever by a doctor before or at 5 years of age.

### Relationship between cohort characteristics and allergic rhinitis.

Table 1 shows the relationship between cohort characteristics and doctor-diagnosed allergic rhinitis by 5 years of age. Factors associated with an increased risk of allergic rhinitis included African-American ethnicity, being born between September and November, maternal sensitization to *Alternaria* (IgE to *Alternaria* > 0.35 U/mL), and having at least one lower respiratory tract illness in the first year of life. Male sex and water damage or mold or mildew in the building of residence within the first year of life were marginally associated with doctor-diagnosed allergic rhinitis by 5 years of age.

In Table 2, we report the number of houses from which each taxon was cultured, and the value corresponding to the 90th percentile. Penicillium was the most common taxon sampled from the air, but Cladosporium, Penicillium, and nonsporulating fungi had the highest levels recovered from the air. From the dust samples, Aspergillus was most commonly recovered sporulating taxon, followed by Cladosporium.

High levels of dust-borne Aureobasidium, Aspergillus, Alternaria, yeasts, and nonsporulating fungi as well as high levels of total fungi were independently associated with the development of doctor-diagnosed allergic rhinitis by 5 years of age in analyses controlling for factors significantly associated with allergic rhinitis (Table 3). These factors included water damage or mold or mildew in the building during the first year of the child’s life, any lower respiratory tract illness in the first year, male sex, African-American race, birth date between September and November, and maternal IgE to Alternaria > 0.35 U/mL.

In a multivariate adjusted proportional hazards regression analysis looking simultaneously at the effects of high fungal levels and significant independent predictors (Table 4), dust-borne Aspergillus [hazard ratio (HR) = 3.27; 95% confidence interval (CI), 1.50–7.14], Aureobasidium (HR = 3.04; 95% CI, 1.33–6.93), and yeasts (HR = 2.67; 95% CI, 1.26–5.66) remained significantly associated with allergic rhinitis. Alternaria and Cladosporium were not significant in the multivariate model, because they are highly correlated with other fungi that were significant. Alternaria was significantly correlated with Aureobasidium (R = 0.33, p < 0.0001) to the point where its contribution to the model is not statistically significant after considering the effect of Aureobasidium. When Aureobasidium is omitted from the model (Table 4, model 3), Alternaria is borderline significant (HR = 2.07; 95% CI, 0.98–4.37). Alternaria and Cladosporium were highly correlated as well (R = 0.30, p < 0.0001). Having high levels of total dust-borne fungi was not significant in multivariate models that had the specific taxa listed above (p = 0.44). The magnitude and strength of these associations remained essentially unchanged when controlling for the presence of dust mite, cockroach, and cat allergens and endotoxin in the home, none of which had significant independent associations with allergic rhinitis by age 5 in multivariate models (data not shown).

## Discussion

High dust-borne fungal levels of Alternaria, Aspergillus, Aureobasidium, and yeasts measured in the home in the first 3 months of life were associated with the subsequent development of doctor-diagnosed allergic rhinitis within the first 5 years of life, independent of water damage or mold or mildew in the home, race, sex, season of birth, lower respiratory tract infection in the first year of life, and maternal IgE to Alternaria > 0.35 U/mL. Home dampness has been shown to be related to allergic rhinitis exacerbations (Kilpelainen et al. 2001), but to our knowledge, the relation of either water damage or mold/mildew or high levels of specific fungal taxa to the development of allergic rhinitis in childhood has not previously been reported.

Although other studies have found associations between measures of total fungi and health outcomes (Gent et al. 2002), it is likely that, in our study, “total fungi” is a noisier estimate of relevant fungal exposures than are our other metrics of fungi. The measure of “total fungi” would combine fungi not likely to be related to rhinitis along with fungi that have been identified as being related to allergic sensitization. This could lead to less precise estimates of fungal heath effects and an attenuation of the relationship between fungi and allergic rhinitis.

Fungal exposure is complex. Fungi can contain allergens, irritants, toxins, and sometimes even potentially infectious units (Johnston 2000). (1→3)-β-d-Glucans—glucose polymers that are structural components of most fungal cell walls—are known to stimulate macrophages and neutrophils and have been shown to be good markers for the overall levels of fungal concentrations in floor dust (Chew et al. 2001). Beijer et al. (2002) found that an inhalation challenge to (1→3)-β-d-glucans has an effect on the inflammatory cells, which may be related to chronic exposure to household molds. Fungi are ubiquitous, and exposure is impossible to completely avoid. Often, there are no adverse effects from these exposures (Macher 2000), but at times exposure to fungi can directly and indirectly influence an individual’s health. Fungal exposure in sensitized individuals may result in histamine and IgE-mediated nasal inflammation (Skoner 2001). Chronic exposure to fungi may also cause chronic nasal symptoms that are not allergic in nature, but rather caused by irritants (Daisey et al. 2003; Dotterud et al. 1996). Symptoms of allergic rhinitis are similar to the chronic nasal symptoms often found with sick building syndrome. Given that fungi may play a role in sick building syndrome as well (Scheel et al. 2001), differentiation may be difficult.

In previous articles we have reported that endotoxin was a risk factor for wheeze (Park et al. 2001) yet had a protective effect on eczema in the first year of life (Phipatanakul et al. 2004). However, the associations of fungi with allergic rhinitis remained essentially unchanged when controlling for the presence of indoor allergens and endotoxin.

There is a large literature demonstrating the relationship between fungal sensitization and allergic rhinitis and asthma, as well as a growing literature linking fungal exposure to asthma and rhinitis exacerbation (Downs et al. 2001; Halonen et al. 1997) in sensitized children (Arshad et al. 2001; Nolles et al. 2001). However, the fact that children with allergic rhinitis are sensitized to fungi does not necessarily mean that fungal exposure will lead to allergy or allergic symptoms. This study suggests a prospective relationship between exposure and symptoms, although elevation of the individual fungal taxa may not lead directly to symptom development. For example, relative elevation of specific taxa such as Aspergillus, Aureobasidium, and yeasts may be a marker for overall fungal exposure. In models containing the individual taxa and our measure of total fungi, total fungi was not predictive of allergic rhinitis, suggesting that the combination of individual taxa was a better marker of clinically relevant fungal exposure.

Sensitization to Alternaria, Aspergillus, and Penicillium allergens have been suggested as being related to allergic rhinitis and asthma exacerbation in children (Clark et al. 1999; Halonen et al. 1997). Purified and characterized allergens have been prepared for Aspergillus fumigatus (Asp f 1 and Asp f 3) (Macher 2000), which has been shown to cause skin reactivity in patients with allergic rhinitis (Johnston 2000). However, A. fumigatus was sampled from only three houses. The most commonly sampled species in this cohort were members of the Aspergillus glaucus group (n = 275) and Aspergillus versicolor (n = 255). Although skin test material is commonly available for both, less is known about the allergenicity of these species. A. versicolor has been implicated in an outbreak of allergic respiratory disease (Jarvis and Morey 2001).

Cladosporium and Alternaria are known sources of allergens, including the proteins Alt a 1, Alt a 2, and Cla h 1 (Johnston 2000). Many cross-sectional studies have correlated Cladosporium and Alternaria sensitization with diagnoses of asthma, eczema, and rhinitis (Arshad et al. 2001; Downs et al. 2001; Halonen et al. 1997; Nolles et al. 2001).

Aureobasidium has been associated with hypersensitivity pneumonitis (Greinert et al. 2000), but its role in the development of allergic rhinitis is unknown. In this study, it may be a marker for mold in the home, rather than the actual source of the chronic nasal symptoms.

In their association with chronic nasal symptoms, yeasts may also be a marker for overall mold exposure, because although they have known associations with skin manifestations of allergy, nasal symptoms are less commonly documented. Yeasts are unicellular fungi that reproduce primarily by budding and, in culture, form pasty colonies similar to those of bacteria (Macher 2000). Several studies have shown that patients with atopic dermatitis and respiratory allergy often develop IgE antibodies against yeasts (Devos and Van der Valk 2000; Nittner-Marszalska et al. 2001; Tengvall Linder et al. 2000). One showed that patients with atopic dermatitis of the head and neck developed IgE antibodies against the yeast Pityrosporum ovale much more often than did their control group. Another found that the enzyme enolase from baker’s yeast (Saccharomyces cerevisiae) induces an immediate dermal allergic reaction in subjects with respiratory allergy and positive skin tests to Candida albicans and other fungi. A third group demonstrated that Pityrosporum orbiculare can induce an eczematous reaction in sensitized atopic dermatitis patients.

### Potential study limitations.

This study does have some limitations. Potential limitations of our work include the relatively small sample size, along with the multiplicity of variables being considered. Our use of the Lasso approach provides an efficient and appealing method for handling multiplicity issues. The Lasso method is much less prone to missing real associations than more traditional adjustments for multiple comparisons, such as Bonferroni. Even so, the reality is that with only 52 cases, some important differences may have been missed. It is unlikely, however, that our analysis erroneously identified many false associations, if any. In addition, because of the collinearity of the fungal data and the paucity of events, it is difficult to assess the risk of each taxon controlling for the others. For example, Alternaria is so significantly correlated with Aureobasidium that its effect is masked when both are included in the model. Even still, in a combined multivariate model, high levels of Aspergillus, Aureobasidium, and yeasts were independently associated with an increased risk of doctor-diagnosed allergic rhinitis within the first 5 years of life. In addition, the air and dust samples were single, short-term samples. Therefore, it is not clear how representative the samples are of long-term household fungal levels. We used only a culture-based analysis to determine fungal concentrations, which would underestimate actual fungal spore exposures and does not account for other fungal agents. Also, the culture media used for both the air and dust samples, DG18, has high solute concentration, which limits the amount of available water. Thus, some strongly hydrophilic fungi (e.g., Stachybotrys sp., some yeasts) might be underrepresented. In addition, all relationships discussed in this article are related to exposures that occurred in the first home in which the child lived, and do not account for conditions in any subsequent homes or locations where the child may have spent significant time (e.g., a child care center). Moreover, without sampling performed throughout the study (Chew et al. 2001), it is impossible to determine whether high fungal levels early in life increase the likelihood of having doctor-diagnosed allergic rhinitis later in life, or whether the early sampling is representative of the environment later in life. In addition, the absence of definitive measurements of allergy, and the relatively early age of ascertainment of outcome in this study such that some children may yet develop allergic rhinitis in this cohort, may be responsible for the lack of an endotoxin effect on the risk of allergic rhinitis in this analysis. Further, parental report of allergic rhinitis or hay fever was not confirmed by chart review, but primary care physician documentation of these diagnoses may not be the gold standard because it is likely to vary by individual practice. In addition, it is possible that the parents did not understand or recall a diagnosis of allergic rhinitis. Lastly, without skin-prick testing, the possibility exists that nasal symptoms related to chronic irritation were misdiagnosed as allergic rhinitis by the physicians of some of the participating children. Even if fungal exposure in infancy leads to allergic rhinitis in some children and to chronic irritant nonallergic rhinitis in others, these findings still have important public health implications.

## Conclusions

Recently, more attention has been given to the need for monitoring of fungal levels and active intervention, where necessary (Johnston 2000). Epidemiologic studies have found associations between report of home dampness and respiratory symptoms in early life, but most have not measured fungi directly and have not determined whether fungal effects can be distinguished from other exposures related to dampness that prospectively predict respiratory disease. Independent of early life report of home dampness, we found that specific dust-borne fungi measurable in the home in the first 3 months of a child’s life was associated with an increased risk of developing doctor-diagnosed allergic rhinitis by 5 years of age.

## Figures and Tables

**Table 1:** Cohort characteristics as predictors of allergic rhinitis.

Factor	No.	Percent with allergic rhinitis	HR	95% CI
Race/ethnicity				
White	309	12.0		
African American	46	21.7	2.22*	1.10–4.47
Hispanic	18	5.6	0.53	0.07–3.82
Asian	26	15.4	1.37	0.49–3.85
Other	6	0	—	—
Season of birth				
Winter (December–February)	103	3.9		
Spring (March–May)	112	12.5	3.33*	1.10–10.12
Summer (June–August)	87	13.8	3.55*	1.15–11.00
Fall (September–November)	103	21.4	5.76**	1.99–16.72
Sex of child				
Female	195	9.7		
Male	210	15.7	1.64	0.93–2.88
Maternal IgE to Alternaria > 0.35 U/mL				
No	316	11.4		
Yes	43	25.6	2.49**	1.27–4.89
Any LRI in year 1				
No	297	10.8		
Yes	108	18.5	1.80*	1.03–3.15
Water damage in year 1				
No	263	11.0		
Yes	135	17.0	1.59	0.92–2.74
Mold or mildew in year 1				
No	246	11.8		
Yes	152	15.1	1.28	0.74–2.22
Water damage or mold				
No	169	10.1		
Yes	235	14.9	1.46	0.82–2.61

Abbreviations: —, no data; LRI, lower respiratory infection.

* p < 0.05;

** p < 0.01.

**Table 2:** Description of fungi included in the analysis.^a^

Fungi	No. > 0	90th percentile
Airborne (cfu/m3)		
Aspergillus	253	100
Cladosporium	315	400
Nonsporulating	303	278
Penicillium	346	189
Yeasts	147	33
Total airborne	402	1,044
Dust-borne (cfu/g)		
Alternaria	233	8,333
Aspergillus	367	33,333
Aureobasidium	322	24,000
Cladosporium	343	33,088
Coelomyces	93	2,941
Fusarium	49	400
Nonsporulating	373	38,889
Penicillium	362	26,087
Ulocladium	60	435
Wallemia	61	1,429
Yeasts	361	58,000
Zygomycetes	57	909
Total dust-borne	405	250,000

a Must have been sampled from the indoor environment and have a 90th percentile > 1 cfu.

**Table 3:** Results of survival analysis of allergic rhinitis.

	Unadjusted		Adjusted^a^	
High fungi level	HR	95% CI	HR	95% CI
Airborne (cfu/m3)				
Aspergillus	1.09	0.44–2.75	1.10	0.43–2.80
Cladosporium	0.72	0.26–2.00	1.25	0.43–3.64
Nonsporulating	0.54	0.17–1.74	0.55	0.17–1.81
Penicillium	1.07	0.42–2.68	0.69	0.23–2.06
Yeasts	0.98	0.36–2.73	0.79	0.24–2.60
Total airborne	0.73	0.27–2.04	0.83	0.28–2.43
Dust-borne (cfu/g)				
Alternaria	2.50**	1.28–4.86	2.34*	1.12–4.91
Aspergillus	2.47**	1.27–4.81	2.57*	1.22–5.40
Aureobasidium	2.77**	1.42–5.39	3.12**	1.50–6.50
Cladosporium	1.63	0.77–3.46	1.88	0.81–4.35
Coelomyces	1.18	0.51–2.77	0.93	0.36–2.38
Fusarium	1.25	0.54–2.94	1.81	0.76–4.34
Nonsporulating	2.21*	1.11–4.41	2.45*	1.15–5.22
Penicillium	1.34	0.57–3.13	1.51	0.63–3.64
Ulocladium	0.82	0.30–2.28	1.04	0.37–2.95
Wallemia	2.23*	1.12–4.45	1.73	0.80–3.75
Yeasts	2.04*	1.00–4.19	2.90**	1.37–6.09
Zygomycetes	0.80	0.29–2.21	0.87	0.31–2.44
Total dust-borne	2.56**	1.32–4.98	3.13**	1.51–6.47

a Controlling for water damage or mold or mildew in year 1, African-American ethnicity, maternal Alternaria IgE > 0.35 U/mL, sex, and birth date in fall.

* p < 0.05;

** p < 0.01.

**Table 4:** Multivariate Cox regression models of allergic rhinitis.

	Model 1^a^		Model 2^b^		Model 3^c^	
Factor	RR	95% CI	RR	95% CI	RR	95% CI
Water damage or mold/mildew in year 1	1.66	0.88–3.15	1.77	0.94–3.34	1.66	0.87–3.17
Male sex	1.79	0.97–3.29	1.88 *	1.03–3.42	1.78	0.97–3.28
LRI ever in year 1	1.58	0.83–3.02	—	—	1.62	0.84–3.12
Maternal IgE to Alternaria > 0.35 U/mol	3.07**	1.49–6.32	2.96**	1.44–6.09	3.21**	1.56–6.60
Race (black vs. others)	3.82**	1.73–8.44	3.79**	1.72–8.38	3.27**	1.51–7.08
Season of birth (Sep–Nov vs. others)	2.44**	1.35–4.42	2.46**	1.35–4.47	2.46**	1.36–4.48
Dust-borne Alternaria	1.40	0.61–3.23	1.52	0.67–3.44	2.07	0.98–4.37
Dust-borne Aspergillus	3.27**	1.50–7.14	2.93**	1.36–6.30	2.73*	1.27–5.87
Dust-borne Aureobasidium	3.04**	1.33–6.93	3.06**	1.35–6.91	—	—
Dust-borne yeasts	2.67*	1.26–5.66	2.80**	1.33–5.93	2.52*	1.18–5.36

Abbreviations: —, not included; LRI, lower respiratory infection.

a Full multivariate model.

b Multivariate model omitting any lower respiratory infection in year 1.

c Multivariate model omitting dust-borne Aureobasidium.

* p < 0.05;

** p < 0.01.
